# Epidemiology of West Nile Disease in Europe and in the Mediterranean Basin from 2009 to 2013

**DOI:** 10.1155/2014/907852

**Published:** 2014-09-11

**Authors:** Daria Di Sabatino, Rossana Bruno, Francesca Sauro, Maria Luisa Danzetta, Francesca Cito, Simona Iannetti, Valeria Narcisi, Fabrizio De Massis, Paolo Calistri

**Affiliations:** Istituto Zooprofilattico Sperimentale dell'Abruzzo e del Molise “G. Caporale,” 64100 Teramo, Italy

## Abstract

West Nile virus (WNV) transmission has been confirmed in the last four years in Europe and in the Mediterranean Basin. An increasing concern towards West Nile disease (WND) has been observed due to the high number of human and animal cases reported in these areas confirming the importance of this zoonosis. A new epidemiological scenario is currently emerging: although new introductions of the virus from abroad are always possible, confirming the epidemiological role played by migratory birds, the infection endemisation in some European territories today is a reality supported by the constant reoccurrence of the same strains across years in the same geographical areas. Despite the WND reoccurrence in the Old World, the overwintering mechanisms are not well known, and the role of local resident birds or mosquitoes in this context is poorly understood. A recent new epidemiological scenario is the spread of lineage 2 strain across European and Mediterranean countries in regions where lineage 1 strain is still circulating creating favourable conditions for genetic reassortments and emergence of new strains. This paper summarizes the main epidemiological findings on WNV occurrence in Europe and in the Mediterranean Basin from 2009 to 2013, considering potential future spread patterns.

## 1. Introduction

West Nile virus (WNV) is RNA virus belonging to the genus* Flavivirus*, Flaviviridae family. Primarily transmitted by the bite of the* Culex *spp. and* Aedes *spp. mosquitoes, WNV is the most widespread member of the Japanese encephalitis virus (JEV) complex [1]. WNV is maintained in nature by a primary transmission cycle between mosquitoes and several bird species, which play the role of amplifying hosts [2, 3]. In the Old World, birds mortality has been sporadically associated with WNV infection [4], as in Israel [5], Hungary [6], and Italy [7]. Humans, horses, and other mammals may be infected by the bite of infected mosquitoes, but they are incidental and dead-end hosts, given the low levels of viraemia that they may develop [8]. Human-to-human transmission may occur only through blood transfusion [9] or organ transplants [10]. Infection in humans is generally asymptomatic, but mild influenza-like symptoms may be observed. In some human categories at risk, like elderly, chronically ill, and immunocompromised people, WNV infection can lead to severe encephalitis and to the death of the patient. In horses the disease course is usually subclinical, although some animals may show neurological symptoms and develop fatal encephalitis.

In temperate countries viral infection in humans and in equines generally occurs in warmer months, from July to October, in accordance with the hypothesis of virus introduction during bird's spring migration followed by the virus amplification in the early summer, contemporaneously with the increase of vector density and influenced by bird population dynamics in nesting geographical areas [11]. The global presence of mosquitoes belonging to the* Culex *genus and the geographical dissemination of WNV by migratory birds underlying the global spread of the infection, especially in tropical and temperate zones.

To date, WNV has been detected both in the Old World (Europe, Middle East, Africa, India, and Asia) and in the New World (North America, Central America, and the Caribbean) and also in Australia (Kunjin virus, a subtype of WNV) [12–14].

Seven distinct genetic lineages of WNV have been described [15], but those two lineages are more frequently recognised: lineage 1, which includes WNV strains circulating in Europe, North America, North Africa, and Australia, and lineage 2, which is historically present in sub-Saharan Africa and Madagascar and more recently observed in some European countries (Albania, Austria, Greece, Hungary, Italy, Romania, Russia, and Serbia). Lineage 2 was considered in the past nonpathogenic for humans and horses [1, 16], but more recently in Europe this strain demonstrated its capacity to cause severe clinical symptoms in both humans and equines [17, 18]. The majority of viruses belonging to lineage 1 are grouped into a cluster called “European Mediterranean/Kenyan cluster,” whereas those responsible for outbreaks in Israel and in the New World are grouped into the “Israeli/American cluster” [19].

Given the rising awareness towards West Nile disease (WND), an increase in the notification of human and equine cases has been observed in the last decades in Europe and in the Mediterranean Basin [20].

Several Mediterranean countries reported WND cases in animals and in humans from 2009 to 2013. During this period, WND cases were reported in Algeria, Bulgaria, Croatia, Former Yugoslav Republic of Macedonia (FYROM), Greece, Hungary, Israel, Italy, Kosovo, Montenegro, Morocco, Occupied Palestinian Territories, Portugal, Romania, Russia, Serbia, Spain, Tunisia, Turkey, and Ukraine.

The reoccurrence of WND cases in the same geographical areas was explained by both the WNV reintroduction by migratory birds and the establishment of overwintering cycles, possibly supported by local bird populations or infected adult mosquitoes surviving during the winter season [21–23]. The aim of this paper is to present the main findings on WNV occurrence in Europe and in the Mediterranean Basin from 2009 to 2013, considering possible trends and potential future further spread patterns.

The data are presented according to lineage strains, although in some cases a presumptive attribution to the most probable lineage has been followed, given the lack of available information on the viral strains involved.

## 2. Occurrence of Lineage 1 WNV Strains from 2009 to 2013

### 2.1. European Mediterranean/Kenyan Cluster

#### 2.1.1. Northern Africa

In 2003, 9 equine WND cases were reported in* Morocco* to be caused by lineage 1 of the virus [19]. No human cases were reported at that time. In August 2010 in the Rabat-Sale-Zemmour-Zaer and Tadla-Azilal regions, respectively, in the north-western and central parts of Morocco, a total of 25 neuroinvasive cases, with 8 deaths, were confirmed in equines in Ben Slimane, Khemisset, Mohammedia, and Casablanca provinces [24, 25]. In 2010, a serological survey in humans identified 11 IgM positive people living near Meknes, Rabat, and Kenitra cities, confirming a recent WNV circulation in the country [26].

After the epidemic occurred in 1994, in October 2012 one WND human case was observed in a 74-year-old man living in France who had travelled in the Jijel province of* Algeria*. He was hospitalized in September with fever and cognitive disorders and died in the same month [27].

Three human cases were reported in* Tunisia* in 2010, in Jendouba and Tataouine regions [28]. WND human cases were also confirmed one year later in three women in Kebili governorate, close to an oasis populated by migratory birds [29]. From August to November 2012, a total of 63 suspected human cases, of which 33 were confirmed, were reported from different governorates: Kebili, Jendouba, Mahdia, Monastir, Bizerte, Sousse, Tozeur, Sfax, and Gabes [30]. In October 2013, 6 human cases were detected in 5 governorates: Gabes, Mahdia, Monastir, Nabeul, and Sousse [31].

#### 2.1.2. Eastern Europe

Clinical cases were never officially reported in* Turkey* up to 2010, although previous serological surveys revealed the human exposure to WNV in various regions [26]. In 2010, 12 laboratory-confirmed cases were detected in humans in 15 provinces located in western Turkey. In 2011, three further laboratory-confirmed cases were detected in the same part of the country. The detection of WNV infections in humans in the same area during two consecutive years may indicate the establishment of a local endemic transmission cycle with virus overwintering [32]. The first isolation of WNV lineage 1 was reported in 2011 in 2 horses in Eskisehir province and in a man in Ankara province [33]. Lineage 1 of WNV was also isolated in 2012 in an 87-year-old woman in Ankara province [34].

In southern* Romania* the first large outbreak of West Nile neuroinvasive disease (WNND) was reported in 1996 [35, 36], albeit the virus circulation was firstly detected in 1955 in central Transylvania and in 1964 in Banat country [37]. After this first epidemic, further investigations confirmed the virus circulation in the country [38]. Lineage 1 strain circulated in Romania between 1997 and 2009 [39]. Serological studies undertaken in 2007 in horses demonstrated the presence of WNV infection in Braila county, in the southeastern part of Romania [40]. From 2008 to 2009, viral circulation was detected in Braila and Dolj counties, where 4 human cases were reported [36]. WNV was detected in* Culex pipiens* females (including overwintering females) collected in Bucharest and Tulcea County from 2007 to 2009 and also in* Coquillettidia richiardii*,* Aedes caspius*, and* Anopheles maculipennis *s.l., collected in the same period in Tulcea County [36].

In October 2010, eight cases were notified for the first time in* Bulgaria*: 5 donkeys and 3 horses bred in the northeastern part of the country were found to be positive to serological tests [41]. No confirmed human cases were detected at that time [28], but in 2012 two human cases in the Burgas oblast region were reported to ECDC [30].

In* Ukraine* 20 human cases of WND were reported between 2011 and 2012 [26]. These cases belonged probably to the same cluster involving Romania and Bulgaria. In August 2013 a further human case was reported in Zhytomyrs'ka oblast [31].

#### 2.1.3. Southern Europe

In* Spain* several WND cases in horses and humans were reported in 2010. The first clinical case was detected in a horse in September 2010 in Andalusia (southern Spain). After the first clinical case a control program for WNV was established and other clinical cases in horses were reported [42]. Forty-four WND cases in horses from Andalusia (in Cádiz, Seville, and Málaga provinces) were notified (Anonymous, 2013). In a study conducted from September to December 2010, fragment of viral RNA belonging to lineage 1 WNV was detected from the blood and the cerebrospinal fluid of a lethally infected horse [43]. In September 2010, the first human case was confirmed in a 60-year-old man and the following month a 77-year-old patient case was reported. Both cases, showing symptoms of encephalitis, were detected in Cádiz in concomitance with WND cases in horses [42]. In 2011 a total of 12 cases in horses in Málaga, Seville, and Cádiz provinces (Andalusia) were notified (Anonymous, 2013), while in 2012 four cases in horses in Cádiz province were documented by the Andalusian authority (Anonymous, 2013). In 2013, a new epidemic involved Seville and Huelva provinces: between August and November 40 cases in horses were confirmed (Anonymous, 2013).

In* Portugal* two WND cases were reported in 2010 in equines, in Lisboa e Vale do Tejo region, showing neurological clinical signs [44].

In* Italy*, in the late summer of 1998, WNV infection was detected for the first time in horses in Toscana region [45]. In August 2008, after 10 years of silence, a large epidemic affected three regions in the northeast of Italy (Emilia Romagna, Veneto, and Lombardy) [46, 47]. In 2009, WND occurred again in the same regions of the previous year and in other regions of central Italy which have never been involved before. A total of 223 cases in equines were confirmed, 37 of which with clinical signs in Emilia Romagna, Friuli-Venezia Giulia, Latium, Lombardy, Tuscany, and Veneto regions. Virus circulation was detected also in birds (the species which was more involved was magpie,* Pica pica*) in Emilia Romagna and Veneto regions, in mosquito pools in Emilia Romagna, and in poultry in Molise region [21]. The phylogenetic analysis of the isolates indicates that the virus circulating in 2009 belonged to lineage 1, with a high identity between 2008 and 2009 WNV Italian strains [21]. This finding strongly supported the hypothesis of virus overwintering and possibly the endemisation in local host populations [21]. In 2010 WNV continued to circulate in the already affected geographical areas, but spreading to new regions, such as Sicily and Apulia regions [48]. A total of 128 equine cases were reported, with 11 of which showing clinical signs. Seroconverted animals were observed in poultry in Molise and Apulia regions. In 2011, additional 197 cases in equines (58 with clinical signs and 14 deaths) were confirmed in the same regions of the previous years, but with the involvement of new areas in southern Italy (Calabria and Basilicata regions), and for the first time Sardinia island. Surveillance in wild bird species in Sardinia allowed the isolation of WNV lineage 1 in a little owl (*Athene noctua*), a jay (*Garrulus glandarius*), and a mallard (*Anas platyrhynchos*). Lineage 1 was detected in Sicily (one mosquito pool and a horse) and in Friuli-Venezia region (one mosquito pool). In 2012, WND was confirmed in 30 horse stables in the same region affected by the virus circulation in the previous years: a total of 63 cases in Veneto, Sardinia, Friuli-Venezia Giulia, and Latium regions were reported, with 15 of which showing clinical signs. Seroconverted sentinel chickens were detected in Basilicata region. Lineage 1 was identified in wild birds and in mosquito pools in Veneto and Friuli-Venezia Giulia regions. No human cases were recorded until 2008. But from 2008 to 2011, 43 WNND cases were reported in five Italian regions (Emilia Romagna, Veneto, Lombardy, Friuli-Venezia Giulia, and Sardinia) with a 16% of case fatality rate [49]. In 2012, 28 WNND cases were identified in the same areas previously affected by WNV infection and in Basilicata region [50]. WNV lineage 1 was identified in blood donors in 2010 and in 2012 in Veneto region. Partial sequencing of the WNV RNA demonstrated an almost perfect identity with the virus isolated in the same area in 2011 in horses and a divergence from the strain responsible for the outbreak in the north of Italy in 2008-2009 [49, 51]. Four human cases occurred in Sardinia at the end of the summer of 2011. The genomic sequences of isolates from three patients revealed a strain strictly related to the WNV strains circulating in Italy in the years 2008 and 2009 and to the strains circulating in Europe and Israel from late 2004 to 2011 [52]. In 2013 WNV circulated in Emilia Romagna, Lombardy, Veneto, Sardinia, and Sicily regions. In Veneto region WNV lineage 1 was detected in an organ donor and in a blood donor [53]. In 2012* Balkan* countries, such as Croatia, Serbia, Montenegro, Kosovo, and the Former Yugoslav Republic of Macedonia (FYROM), reported WNV human cases. For Croatia, Kosovo, Serbia, and Montenegro that was the first notification of WNV infection in humans.

*Croatia* reported 5 cases in humans and 12 cases in equines without apparent clinical signs from July to August 2012. Both equine and human cases occurred in the eastern part of Croatia [30, 54]. In 2013, 16 human cases, of which one was confirmed, in Medimurska, Zagreb, and Zagrebacka areas [31] were identified.

In 2012,* Kosovo* and* Montenegro* reported, respectively, 6 and 1 human cases [26]. In 2013 in Montenegro four additional human cases were notified [31]. In 2011* FYROM* reported 4 confirmed human cases in Skopje, occurring from August the 25th to October the 6th, and additional 10 confirmed cases in horses and 36 in birds [55]. In 2012, six further human cases were reported in FYROM [30]. In 2013 a human case was identified in July [31].

For the first time, WNV human cases were reported in* Bosnia-Herzegovina* in 2012 [26]. In 2013, 3 human cases were confirmed in Modrica and Tuzlansko-Podrinjski cantons [31]. Between late August and early September 2013 WNV infection has been detected in 2 hooded crows (*Corvus cornix*) [56].

#### 2.1.4. Western Europe

In* France*, after the cases reported in 2003 and 2004 in humans and horses, 4 distinct foci of WND were reported: WNV was responsible for neurological syndromes in horses of Camargue region between 2000 and 2004 and between 2003 and 2006 in the Var and Eastern Pyrenees Departments [57].

### 2.2. Israeli/American Cluster

#### 2.2.1. Middle East

In 2000,* Israel* experienced its largest WNF epidemic with 429 reported human cases. After this epidemic, 68 neuroinvasive human cases were reported in 2010, 36 in 2011, and 63 in 2012 [58]. In 2011 WNV lineage 1 was isolated from a mosquito pool [59]. In 2013, 63 human cases were documented in Central, Haifa, Southern, and Tel Aviv districts [31].

**Figure 1:**
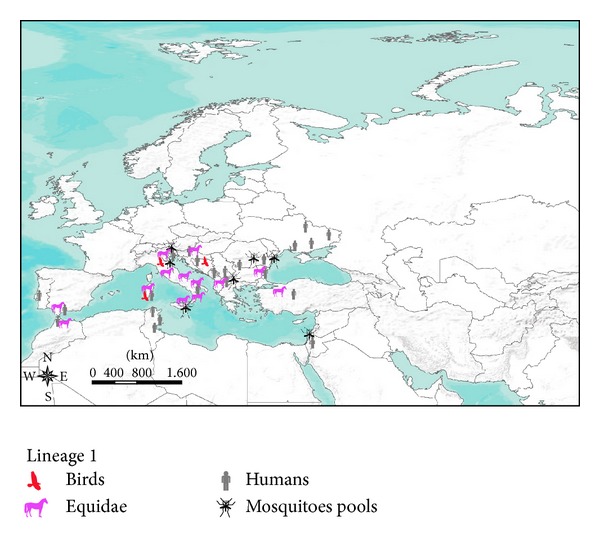
Lineage 1 strain occurrence in Europe and in the Mediterranean Basin from 2009 to 2013.

In Figure 1 a comprehensive map of WNV lineage 1 occurrence in Europe and in the Mediterranean Basin from 2009 to 2013 is represented.

## 3. Occurrence of Lineage 2 WNV Strains from 2009 to 2013 

### 3.1. Eastern Europe

Until 2004 lineage 2 WNV was not detected outside of Africa, but from this year lineage 2 was repeatedly identified in several parts of Europe. In 2004, a WNV strain correlated to the Central Africa lineage 2 viruses was isolated from goshawks (*Accipiter gentilis*) in southeast* Hungary*. Sporadic cases of infection were observed in this country between 2004 and 2007 in wild birds, sheep, horses, and humans. In 2008 and 2009 lineage 2 WNV strain was detected in Hungary and* Austria*, where the virus was isolated from wild hawks (*Accipiter *spp.) and one captive kea (*Nestor notabilis*) [18, 60]. After 2008, human cases were notified in Hungary from 2010 to 2012: 3 cases were reported in 2010, 1 of which in a man living close to the Romanian border, 3 cases in 2011, and 17 in 2012 [30]. In 2013, 31 human cases were reported between September and October [31].

In* Russia*, large epidemics of WND in humans were observed in the area of Volgograd since 2007, when RNA of WNV belonging to lineage 2 was detected in human brain and blood samples. In 2010 the same viral strain was responsible for a total of 552 human cases in Russia [61, 62], which represents the largest number of WND human cases that has never been registered in that country before. In addition to Volgograd province, other regions were involved: Rostov, Voronezh, Krasnodar, Astrakhan, Kalmoukia, Tatarstan, and Chelyabinsk oblasts [26]. Volgograd province was the most affected area by the viral circulation also in 2011, with 61 cases out of a total of 153 [26]. In 2012, 447 human cases (210 of them in the Volgograd province) were notified in Russia [30].

In 2013, 177 human cases were confirmed in Russia (Astrakhanskaya oblast: 69 cases; Volgogradskaya oblast: 49 cases; Saratovskaya oblast: 30 cases; Adygea Republic: 1 case; Belgorodskaya: 2 cases; Kaluzhskaya: 1 case; Lipetskaya: 2 cases; Omskaya: 1 case; Orenburgskaya: 1 case; Rostovskaya: 8 cases; Samarskaya: 9 cases; and Voronezhskaya oblasts: 4 cases [31].

In* Romania*, an apparent change in the epidemiological situation was observed in 2010: for the first time in more than ten years, several human confirmed cases were detected also in the central and northern provinces of the country, not reached before by the infection [36]. In 2010 molecular investigations revealed that these episodes of WNV infection were due to lineage 2, genetically related to the 2007 Russian strain [35]. A total of 83 human cases were reported between 2010 and 2012 [26]. In 2010 human cases were distributed in 19 districts all over the country, with clusters of infection in the southeastern district of Constanta and in the urban areas of Blaj (in the western Romania) and Bucharest. Many cases (*n* = 35) were recorded in the southern part of the country, which is an area known as having been endemic for WNV during previous years. However, WNV infection was reported in humans in previously unaffected areas, such as districts in central Transylvania and in the Moldavian Plateau [35]. WNV circulation was observed also in horses. In 2010, 6 cases of equine infection were notified to OIE: 5 cases in Braila and one in Constanta County, in southeast Romania [63]. In 2011 and 2012, most of the cases were recorded in Bucharest urban area [26].

From August to October 2013, 24 human cases were reported from different municipalities of Romania: Bacau, Braila, Bucharest, Constanta, Galati, Ialomita, Iasi, Ilfov, Mures, Sibiu, and Tulcea [31].

### 3.2. Middle East

In* Israel* lineage 2 was detected between 2009 and 2010 in mosquito pools collected in the northern part of the country [59].

### 3.3. Southern Europe

In the summer of 2010, 261 human WNV infections were diagnosed for the first time in* Greece*, including 197 neuroinvasive cases and 34 deaths. Most cases occurred in the northeastern part of the country [64]. Lineage 2 WNV strain was detected in* Culex pipiens* mosquitoes collected in two locations where human cases were reported, in one blood donor living in the same area and in resident birds (*Eurasian magpie*) [65–68]. WNV lineage 2 genomic sequences obtained from viruses isolated from one affected person [68] and from mosquito pools [64, 65, 69] showed a high genetic identity to the Hungarian WNV strain isolated from birds in 2004 [70]. In 2010, additional 30 cases in equines were confirmed [71]. One year later, in 2011, 101 human cases (with 8 deaths) were reported in Greece. The infection spread to new areas and 17 further cases occurred in districts that had not been previously affected [72]. Twenty-three equine cases (with 1 death) were confirmed in 2011 [73]. Genomic sequences of the virus were obtained from a seroconverted chicken in July 2011 in the city of Agios Athanasios [74]. This isolate showed a close genetic relationship with lineage 2 strain which emerged in Hungary in 2004 as well as a high homology with the Nea Santa strain detected in* Culex pipiens* in 2010 in Greece [74]. In 2012, a total of 161 human cases were reported, but only 47 were confirmed by laboratory investigations [75]. In the same year, 15 equine cases were notified and confirmed [76]. The molecular characterization of two isolates from chickens suggested that the virus responsible for the epidemic in Greece in 2012 was again the Nea Santa-Greece-2010 strain [77]. In 2013, for the fourth consecutive year, Greece reported WNV infection in humans: 86 cases were confirmed from the regional units of East Attica, Athens, Thessaloniki, Imathia, Xanthi, Kavala, Serres, Corfu, and Pella, already affected by the virus circulation in the previous years, and the newly infected region of Ileia [78]. In addition, 15 horse cases were reported in Xanthi, Attiki, Achaia, Kavala, Evros, Serres, and Lasithi [79].

During 2011 in* Italy* the WNV caused several outbreaks among horses and birds. Lineage 2 strain was found in two pools of* Culex pipiens *collected in Friuli-Venezia Giulia region and in the tissues of a resident collared dove (*Streptopelia decaocto*) found dead in Veneto region, in northeast Italy [80]. During the summer of 2011, WNV lineage 2 was also detected in urine samples of a febrile patient in Marche region [81] and in a patient coming from northeastern Sardinia. These strains were closely related to each other and to those responsible for the outbreaks that occurred in Greece and Hungary in 2010 and 2005, respectively [52, 69].

Two mosquito pools (*Culex pipiens*) collected in 2012 were found positive to WND lineage 2 in Veneto and Sardinia [82] and in the same year WNV strains belonging to lineage 2 were detected and isolated from the tissues of goshawk (*Accipiter gentilis*) and carrion crows (*Corvus corone*) in Sardinia [7].

In 2013 the presence of 50 cases was confirmed in horses, 12 of which were clinical, in Veneto, Lombardy, Emilia Romagna, Calabria, Sardinia, and Sicily regions. The analysis, of one dead horse in Emilia Romagna, confirmed the circulation of WNV lineage 2. Lineage 2 circulation has been confirmed in mosquitoes and in wild birds in Veneto, Lombardy, Emilia Romagna, and Sardinia regions [82].

In 2013 40 neuroinvasive cases of WND (WNND) have been reported in humans in Veneto, Emilia Romagna, Lombardy, and Apulia regions and 30 people with West Nile fever tested positive to WNV in Veneto, Emilia Romagna, and Lombardy regions. Lineage 2 was identified in Veneto region in plasma and/or urine of seven patients with WNND or WNF and in a blood donor, while WNV lineage 1 was, respectively, detected in an organ donor and in a blood donor [53]. Therefore in 2013 the cocirculation of lineages 1 and 2 has been confirmed in Veneto region in mosquitoes and human [53, 82].

In* Albania* a human case was confirmed in 2010 in a 14-year-old child in the southeast prefecture of Korce (bordering Greece). In 2011, 49 human cases (15 confirmed) of WNV infections were detected in the coastal and central parts of Albania. Lineage 2 was confirmed to be the causative agent of human cases reported in 2011 [26].

In 2012, WNV infection was described in animals in Albania. In a study performed in 2012, 37 out of 167 collected equine sera were positive to serological tests, while no WNV-specific antibodies were detected in 95 samples from domestic birds [83].

In* Serbia*, in 2009 and 2010, 349 horses were randomly collected in Belgrade (in Sabac and in Vojvodina regions) and analysed for WNV-specific neutralising antibodies. This study reported the first serological evidence of WNV infection in Serbia: 42 (12%) seropositive horses were detected [84].

In 2012, 71 human cases were notified, with 53 of which in Belgrade. This was the first reported episode of WNV infection in humans in Serbia [26]. Antibodies against WNV were detected in 7 samples collected from wild bird species (four from mute swans (*Cygnus olor*), two from white-tailed eagles (*Haliaeetus albicilla*), and one from a common pheasant (*Phasianus colchicus*) in 2012 in Vojvodina [85]. Nine WNV RNA positive birds, three northern goshawks (*Accipiter gentilis*), two white-tailed eagles, one legged gull (*Larus michahellis*), one hooded crow (*Corvus cornix*), one bearded parrot-bill (*Panurus biarmicus*), and one common pheasant, were detected. The phylogenetic analysis showed two distinct clusters of lineage 2 closely related to those circulating in neighbouring countries (Greece and Hungary) [85]. This was the first report of the occurrence of WNV in wild birds in Serbia [85]. Entomological investigations performed in Belgrade in August 2012 revealed the presence of WNV lineage 2 nucleic acid in 10 mosquito pools [70, 86]. In 2013, 302 human cases were reported between July and October, with the majority of them concentrated in Grad Beograd area [31].

**Figure 2:**
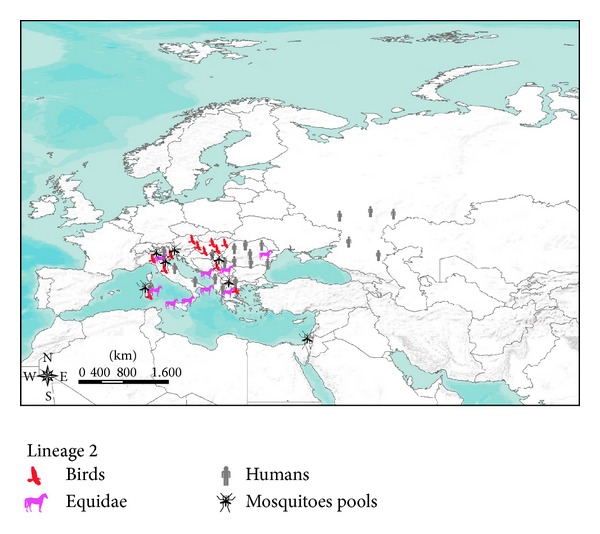
Lineage 2 strain occurrence in Europe and in the Mediterranean Basin from 2009 to 2013.

Figure 2 shows the geographical distribution of WNV lineage 2 strain in Europe and in the Mediterranean Basin from 2009 to 2013.

## 4. Discussion and Conclusions

The presence of WNV in the Old World is well known since decades. WNV was first identified in 1937 from a native woman of the West Nile province of Uganda [87]. Since then, both sporadic cases and major outbreaks of WND were reported in Africa, Middle East, Europe, and Asia. Epidemiological aspects of WNV transmission were well documented in the early 1950s in Egypt and in Israel, in the 1960s in France, and in the 1970s in South Africa [4, 13, 88].

In Europe the first significant urban epidemic occurred in Bucharest (Romania) in 1996 [37]. Since then the WNV sporadically occurred around the Mediterranean and Eastern European countries. During the last two decades, however, the disease reemerged in Europe with an increasing frequency in humans, where severe cases of neuroinvasive disease were observed. The WNV detection in geographical areas apparently not previously affected by virus transmission, the severity of the infection in humans and horses, the absence of an effective vaccine to protect people, and the mosquito-borne transmission give to this disease all the characteristics required to be considered a major threat for public health in many countries.

The increased number of cases of WNV infection notified in Europe and in the Mediterranean Basin in the last years may be partially due to the rising awareness about this infection, but an actual spread of the virus across the Mediterranean and European countries cannot be excluded, and the possible consequences of this increased exposure to the virus for human populations should not be underestimated.

The transmission of WNV across Europe and Mediterranean Basin currently depicts new scenarios. The WNV circulation in Europe is probably greatly influenced by the flyways of migratory bird species. It is noteworthy that some territories of northwestern Europe were never affected by virus circulation. The United Kingdom, the northwest of France, The Netherlands, Belgium, Denmark, Germany, and Scandinavian and Baltic countries apparently did not experience any case of WNV infection in the past (Figures 1 and 2). This apparent difference of WNV occurrence in Europe cannot be explained simply by a different mosquito fauna composition or abundance. The known European spatial distribution of some vector species, like* Ochlerotatus caspius* or* Culex pipiens*, does not support a different susceptibility of northwestern Europe to WNV infection [89]. The actual knowledge may reasonably suggest a more decisive role of migratory birds in the spread of WNV across the European continent [90]. However, a better evaluation of the influence of bird migratory routes on the spatial spread of WNV in Europe would be fundamental for a more accurate assessment of the risk of WNV introduction into new areas.

On the other hand, the WNV is constantly detected in several territories of the Mediterranean Basin and in southeastern Europe. This constant WNV occurrence in the Mediterranean region cannot be caused only by the virus reintroduction from the sub-Saharan Africa. Probably endemic cycles are established in the Mediterranean area, although it is not clear which bird species might play a role in the local persistence of the infection or the contribution of overwintering mosquitoes. In addition, the role of persistent infection in organs of infected birds cannot be excluded, which may represent a further infection overwintering mechanism when coupled with the predation by other bird species, as, for example, those belonging to Corvidae or Falconidae families [91].

A relevant epidemiological finding in the recent years is represented by the spread of lineage 2 across Europe and Mediterranean Basin, with zones where the circulation of the two lineages coexists. To date the copresence of the two lineages has been proven in Italy and in Romania (Figures 1 and 2).

Lineage 2, which was endemic in sub-Saharan countries of Africa, was firstly identified in Hungary in 2004, then in Russia in 2007, in Romania in 2010, and in Italy in 2011 [1]. The genetic homology of lineage 2 detected in Hungary in 2004 with the Greek isolates in 2010 [69], with the Italian strains circulating in 2011 and 2012 [7], and with isolates in Serbia 2012 [85] suggests the involvement of wild birds species able to spread the virus in wide areas of the Mediterranean and Balkan areas.

In Italy lineage 1 seems to be more linked to large epidemics, especially in areas surrounding wetlands with a significant population of migratory birds (e.g., the delta of Po River or wetlands in Sardinia island), whereas lineage 2 sporadically occurs in scattered locations across the country, without causing apparent large epidemics. Similarly, WNV lineage 2 has been sporadically detected in birds in Austria, whereas a considerable number of human cases were associated with the circulation of this lineage in Romania, Hungary, Greece, and Russia.

Possible differences between lineage 1 and lineage 2 viruses in their pathogenicity for birds have been poorly investigated [91]. In 2013, through an experimental study, Ziegler et al. proved the high virulence of WNV lineage viruses 1 (isolated in New York in 1999) and 2 (strain isolated in Austria in 2009) in falcons showing no significant differences in mortality rates or viraemia levels [92].

Similarly little information is available on possible different characteristics of lineages 1 and 2 viruses in relation to the vector competence of the main mosquito species. The unique available study compares the vector competence of African vectors (*Culex neavei *and* Culex quinquefasciatus*) for different African WNV lineages [93] and, therefore, its results cannot be extrapolated outside the African continent.

WNV lineage 2 isolated in Italy is genetically related to those detected in Hungary [7, 94] and, therefore, the apparent dissimilar capacity of spreading of this lineage between Italy and other countries cannot rely on virus diversity but probably is due to local ecological and epidemiological conditions. In particular, the sequence analysis of WNV lineage 2 isolated in Sardinia Island from a northern goshawk (*Accipiter gentilis*) suggests a common origin with Hungarian isolates, thereby supporting a role of short-range migratory birds in the spread of virus in Italy from central Europe. According to this hypothesis, the differences observed in Italy between the spatial distribution and occurrence of the two lineages would be linked exclusively to a more recent introduction of lineage 2 from central and eastern European countries, where this virus is endemic [7, 94]. In addition, the observed differences in the amino acids composition of viral NS3 protein between Greek lineage 2 isolates and the Italian ones could explain the higher virulence of Greek strain for humans [7, 94].

The cocirculation of lineages 1 and 2 in some countries and the genetic variation between strains isolated in different years in the same country [21, 95] may create the favourable conditions for genetic reassortments with possible variations in the virulence of the viral strains, which would lead to consequences presently difficult to assess.

In addition, the influence of possible changes of climatic and environmental conditions should not be underestimated in the observed spread of WNV in the European and Mediterranean countries. These factors, in fact, may influence the seasonality of disease transmission [96], due to increased number of mosquito replication cycles (consequently also a higher rate of overwintering virus-carrying mosquitoes) and increased virus transmission rates [97].

Given the absence of evidence for vertical transmission in mosquitoes (although it cannot be excluded), the persistence of the infection due to the survival of infected adult mosquitoes during winters or a so-far unidentified vertebrate reservoir host is hypothesized for being responsible for the maintenance of the virus. Resident birds seem to be particularly suitable for this role, given their density and the ability for some species to fly for relatively long distances, independently of seasonal migratory pattern.

Since 2010, WNV showed a clear capacity both to spread into areas not previously affected by the viral circulation and to persist in areas where the ecological and climatic conditions are favourable to its circulation.

A further aspect to be considered is the cocirculation in the Old World of WNV and other flaviviruses, sharing the same hosts and ecological niches, as for the Usutu virus. In Italy the circulation of Usutu virus has been detected simultaneously with WNV in several geographical areas [98], with the possibility of recombination, which may influence the transmission capacity and the occurrence of these viruses in vertebrate hosts.

In the last decades WNV continued to evolve, changing its transmission rate and geographical patterns. The adaptability showed by this multihost virus should induce all researchers to continuously and carefully monitor the evolution of the epidemiological situation of WND in Europe and in the Mediterranean Basin.

Further studies would be useful also to fill some existing gaps in our current knowledge on WNV epidemiology. For such reasons, public health and veterinary officials should strictly cooperate to establish effective early warning systems across the region, useful to prevent and reduce the impact of this emerging disease on human and animal health.
